# Lameness in piglets – should pain killers be included at treatment?

**DOI:** 10.1186/s40813-016-0022-5

**Published:** 2016-03-08

**Authors:** Mate Zoric, Ulla Schmidt, Anna Wallenbeck, Per Wallgren

**Affiliations:** 1 Department of Animal Health and Antimicrobial Strategies, National Veterinary Institute, SE-751 89 Uppsala, Sweden; 2 Department of Clinical Sciences, Faculty of Veterinary Medicine and Animal Sciences, Swedish University of Agricultural Sciences, Box 7054, SE-750 07 Uppsala, Sweden; 3 Department of Animal Breeding and Genetics, Swedish University of Agricultural Sciences, Box 7023, SE-750 07 Uppsala, Sweden

**Keywords:** Piglets, Lameness, Treatment, Penicillin, Non Steroidal Anti-Inflammatory Drugs, NSAID

## Abstract

**Background:**

Joint swelling and lameness are the most obvious and persistent clinical signs of infectious arthritis in piglets. For a positive treatment effect of piglets with arthritis, early initiated treatments with antibiotics are desired. Hitherto pain-reducing drugs have rarely been used within veterinary medicine, but the potential of non steroid anti-inflammatory drugs (NSAID) are interesting from an animal welfare perspective. Therefore, the aim of this study was to compare the long term efficiency of treating lameness with and without pain relief. Further, the incidences of affected joints in lame piglets were analysed.

**Results:**

In total 415 of the 6,787 liveborn piglets included in the study were diagnosed with lameness (6.1 %). Around 86 % of these diagnoses took place during the first 3 weeks of life. There was no difference in the incidence of lameness between the sexes, but lameness was most commonly diagnosed in the offspring to old sows (>4 parturitions). Lameness was diagnosed in about every second litter and on average about two pigs were diagnosed in the affected litters. The incidence of affected litters as well as affected piglets increased with ageing of the sows.

Treatments with antibiotics solely and in combination with NSAID improved (*P* < 0.01 to 0.001) the clinical status from day to day, but the clinical response did not differ between the two treatment groups.

Piglets that remained healthy were 1.1 and 1.7 kg heavier (*P* < 0.001) than piglets diagnosed with lameness at 5 and 9 weeks of age, respectively. There were no differences in piglet body weights between the treatment strategies at any time.

**Conclusions:**

The clinical response to penicillin was good. It was neither improved nor reduced by a concurrent administration of NSAIDs. Nevertheless NSAIDs may improve the animal welfare due to pain relief. An important finding of this study was that decreasing pain due to lameness not was negative in a long term perspective, *i.e.* reducing pain did not lead to overstrain of affected joints and no clinical signs of adverse effects were noted. Therefore the use of NSAIDs ought to be considered to improve the animal welfare, at least in severe cases.

## Background

Abrasions, wounds and necrosis in the skin or on the hooves and accessory digits, are very common in newborn piglets [1]. Risk factors include floor type, nutrition and genetics [2, 3]. Skin lesions in piglets are presumably mainly a result of contact with the floor, especially during suckling [4–8]. The lesions are generally bilateral and most commonly observed as abrasions over the carpal joints [9, 10]. Such lesions are present already on day 3, they increase in magnitude until day 10 and thereafter decline [6, 8, 11]. Foot and skin lesions can contribute to lameness in two ways, either due to pain induced by the injury itself or by acting as an entrance for infections that spread to joints through bacteraemia and thereby induce arthritis and pain [2, 12]. Infectious arthritis are dominated by hemolytic streptococci, but also staphylococci and *E. coli* are frequently demonstrated [6, 8, 12, 13]. The streptococci domination suggests the sow to be a significant source of infection to the piglets [13, 14]. Lameness in suckling piglets is observed in about every second litter and around 75 % of the treatments against lameness are effectuated in piglets less than 3 week of age [12, 15, 16]. Apart from animal suffering, lameness contributes to losses in terms of dead piglets, decreased growth an increased use of manual labour and of antibiotics [16, 17].

Pain may of course be transient, but if the recovery period is prolonged the animal will be less competitive, e.g. at group feedings situations. If the pain cannot be effectively treated, culling may be the only practical option in pig farming [18]. Thus, the therapy of lame piglets ought to include measures aimed to decrease pain and thereby also minimize any adverse effect on feed intake [19].

Historically, little emphasis has been paid on pain management in veterinary medicine [20]. Pain has been regarded as a tool to keep animals tranquil to allow any injury to heal faster. Knowledge of pain management has been limited, both among veterinarians in the academic environment and in clinical practice [21]. However, supporting therapy with analgesic drugs (NSAIDs = Non Steroidal Anti-Inflammatory Drugs) has increased considerably in recent years [22], explained by a greater awareness and understanding of pain and painful conditions [23]. NSAIDs have anti-inflammatory, analgesic and antipyretic effects [24]. They have mainly a peripheral analgesic activity and acts by inhibiting the synthesis of prostaglandins, which in turn sensitivities nocisceptores (peripheral sensory nerve endings that react strong to tissue thermal, mechanical and chemical stimuli). Ketoprofen (2-(phenyl 3-benzoyl) propionic acid) is a NSAID of the 2-arylpropionic acid group (generically known as profens) with analgesic, anti-inflammatory and antipyretic properties [18].

To reduce pain in piglets, NSAIDs is at present the only realistic alternative since drugs of this class are the only long-acting analgesics with maximum residue limits (MRL) established for pigs in Europe [18]. However, as the analgesic is administered by intramuscular injection, treatment of large numbers of piglets have been concluded to be time consuming and potentially costly [25]. Further, if analgesic treatment of lame piglets leads to an increased mobility with the risk for over-load of affected joints with side effects in the future cannot be excluded. Therefore, the aim of this study was to investigate the clinical effects of concurrent treatment of lame piglets with NSAID-drugs and antibiotics to that of using antibiotics solely.

## Results

### Relationship to lameness and age of piglets

In total 415 out of 6,787 liveborn piglets were diagnosed with lameness (6.1 %) during the two and half years studied. Around 86 % of these diagnoses took place during the first 3 weeks of life and the risk incidence of lameness decreased from 2.4 % during the first week of life to 0.3 % during the fifth week of life. There was no difference in the incidence of lameness between the sexes (Table 1).

**Table 1:** The mean incidence risk for being diagnosed for lameness with respect to week of age in 6,787 live born piglets

Age (Weeks)	Piglets medically treated for lameness (*n*)	Incidence risk of lameness (%)
1	166	2.4
2	119	1.8
3	72	1.1
4	35	0.5
5	23	0.3
In total	415 of 6,787	6.1
Whereof males	215 of 3,553	6.1
Whereof females	200 of 3,234	6.2

### Relationship with lameness and parity of sow

Overall, lameness was diagnosed in about every second litter, but the range of lame piglets varied from one to nine in affected litters (Table 2). The incidence of lameness was lowest in the litters of first and second parity sows and then increased with the age of the sows, both with respect to affected litters and to affected piglets within litter.

**Table 2:** Prevalence of litters with lameness diagnosed in piglets by sow parity. Total prevalence, mean number and range of lame pigs in the affected litters, as well as the percentages of litters with one, two, three or more than three affected piglets

	Litters	Litters with lameness		Number of lame piglets in affected litters					
Parity	Total	*n*	%	Mean	Range	1 lame	2 lame	3 lame	>3 lame
1	146	46	31.5 ^A^	1.52	1-9	76 %^A^	15 %^A^	4 %^A^	4 %^A^
2	103	39	37.9 ^AB^	1.51	1-6	74 %^AB^	18 %^A^	5 %^A^	5 %^A^
3	97	40	41.2 ^AB^	1.74	1-6	55 %^BC^	28 %^A^	8 %^A^	8 %^AB^
4	62	33	53.2 ^B^	2.15	1-6	48 %^C^	51 %^B^	15 %^AB^	15 %^AB^
>4	73	52	71.2 ^C^	2.18	1-7	54%^C^	29%^A^	27%^B^	19%^B^

Different superscript letters within columns indicate significant pairwise differences with *P* < 0.05

### Clinical effect of treatments

**Fig. 1:**
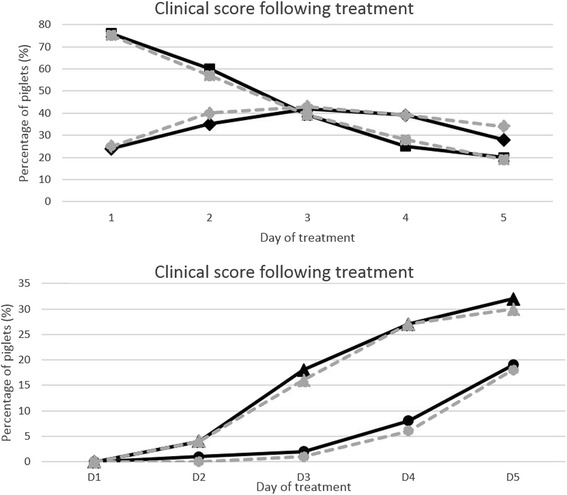
The clinical score of lame piglets following treatment initiated on day 1. The clinical score of lame piglets treated with penicillin solely (grey dotted lines) compared to pigs treated with penicillin and NSAID (black lines). The decreasing prevalence of lame pigs with severe signs (diagnose code 3; squares) initially increased the prevalence of pigs with major clinical sigs (diagnose code 2; diamonds) from 25 to 40 %, but at day 5 also the prevalence of this diagnose had ceased somewhat (top). As a consequence, increasing levels of almost healthy (diagnose code 1; triangles) or healthy piglets (diagnose code 0; circles) were denoted (bottom). Note the different scales on the y-angles

Both treatment strategies, with penicillin solely or with penicillin in combination with NSAIDs, improved (*P* < 0.01 to 0.001) the clinical status (i.e. improved lameness score) from day to day but the treatment efficacy did not differ between the groups. Approximately 75 % of the piglets diagnosed with lameness was scored with severe signs of lameness (score 3) at the onset of treatment while 50 % were scored healthy or almost healthy (score 0 or 1) day 5 of treatment. The treatment efficacy is illustrated in Fig. 1, showing the day to day prevalence of piglets within the different diagnose codes.

### Relationship to lameness and weight

The weights recorded at birth, 5 and 9 weeks of age are shown in Table 3. Piglets that remained healthy were 1.1 and 1.7 kg heavier than piglets attended with lameness at 5 and 9 weeks of age, respectively. Piglets attended for lameness performed equal regardless of treatments strategy (Fig. 1), but piglets that remained free from lameness grew 9 % faster (*P* < 0.001) than piglets diagnosed with lameness.

**Table 3:** Mean weights of piglets treated for lameness during the first 9 weeks of life compared to piglets not attended with lameness. Every second lame piglet was treated with penicillin and NSAID and every second piglet was treated with penicillin solely. Least Square Means ± Standard Error

	Unaffected	Treated for arthritis		Treated with	Treated with	
		All		Penicillin + NSAID	Penicillin	
	(kg)	(kg)	*P*	(kg)	(kg)	*P*
Birth	1.5 ± 0.01 (*n* = 6373)	1.6 ± 0.02 (*n* = 412)	n.s.	1.5 ± 0.03 (*n* = 207)	1.6 ± 0.03 (*n* = 205)	n.s.
5 weeks	10.6 ± 0.11 (*n* = 4804)	9.5 ± 0.14 (*n* = 372)	<0.001	9.0 ± 0.20 (*n* = 184)	9.1 ± 0.20 (*n* = 188)	n.s.
9 weeks	24.3 ± 0.19 (*n* = 4161)	22.6 ± 0.26 (*n* = 354)	<0.001	21.7 ± 0.37 (*n* = 182)	22.4 ± 0.37 (*n* = 172)	n.s.

n.s. = not significant, *P* > 0.05

### Distributions of the affected joints

A total of 454 joints were associated with lameness in 415 affected piglets. One clinically affected joint was recorded in 380 piglets (91.5 %), two joints in 31 piglets (7.5 %) and three joints in 4 piglets (1 %).

**Fig. 2:**
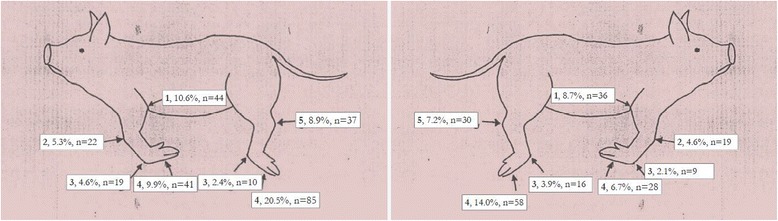
Which joints are affected? The affected joints in suckling piglets attended with lameness are numbered and have name as follows; 1. Elbows; 2. Carpus; 3. Metacarpal joints; 4. Hoofs; 5. Hocks. The prevalence and total of affected joints on the left side of affected piglets to the left and on the right side of affected piglets on to the right

The distribution of the affected joints is shown in Fig. 2. It was evenly distributed between front and hind legs with 52.5 % in the front legs (Elbows 19.3 %; Carpus 9.9 %; Front Metacarpal joints 6.7 %; Front Hoofs 16.6 %) and 56.9 % in the hind legs (Hocks 16.1 %; Back Metacarpal joints 6.3 %; Back Hoofs 34.5 %).

In total, 56.8 % (*n* = 258) of the lesions were recorded on the left side of the piglets and 43.2 % (*n* = 196) on the right side.

### Necropsies, bacteria and antimicrobial resistance

Three lame piglets were culled before medical treatment and subjected to necropsy, including histopathological and microbiological examinations. One of the three pigs suffered from acute purulent arthritis, the other two of chronic arthritis, and all of the piglets were affected in more than one joint. Bacterial cultivations of three joints per animal demonstrated microbial growth in all piglets. The findings were *Streptococcus dysgalactiae* subsp. *equisimilis* in one piglet and *Staphylococcus hyicus* subsp. *hyicus* in two piglets. They were all sensitive to all antibiotics included in the in the [VetMIC™ GP-mo-A (version 2), National Veterinary Institute, NVI].

## Discussion

This study was conducted at a research station with experienced staff that had good recording systems and written instructions for diagnosing diseases. Lameness was defined as lameness and/or swollen joint(s), thereby not differentiating lameness due to infections from lameness due to other causes. However, in a previous study where lame piglets were euthanized instead of medically treated, the diagnose arthritis was always made at necropsy [6, 8, 12], as also was the case with the three piglets sacrificed in this study. Thereby it is believed that most lame piglets actually suffered from arthritis, but as no etiological diagnose was made in the other piglets we prefer to use the term lameness.

During the two and half years studied, 415 of the 6,787 piglets born had been treated for lameness before the age of 5 weeks (6.1 %), whereof 86 % were diagnosed within 3 weeks from birth. Skin cuts have been discussed as an entry for infections, and castration may therefore predispose for lameness. However, as no difference in the incidence of lameness between the sexes were recorded, the results obtained concur previous reports [16, 26, 27], suggesting that castration in itself appear not to predispose to development of lameness - provided that it is effectuated skilfully and under aseptic conditions. Instead there was a correlation to the age of the sow, lameness was most commonly diagnosed in piglets born by old sows (>4 parturitions).

Lameness in piglets is of concern for both animal welfare and economic reasons. In intensive pig production the weight of the weaned piglet has a significant influence on lifetime performance. Low weight at weaning implies a loss of income for the farmer and might also influence the welfare of the affected animals negatively. Lameness, as well as other diseases [28], reduce the growth rate of the piglets and the piglets that were treated for lameness in this study grew 9 % slower than those not diagnosed with lameness. In a Danish study, piglets treated for lameness, diarrhoea or other infections were identified as main contributors to a decreased weight gain during the suckling period, with 38, 8 and 21 g per day, respectively [29].

Lame piglets are also believed to suffer from pain and stress, which is reported to have a negative influence on production [3]. Pain is defined as an unpleasant sensory and emotional experience that is associated with actual or potential tissue damage [30]. However, pain is subjective and therefore difficult to quantify, and there are no specific parameters for measuring pain [31]. Nevertheless, it is widely accepted that piglets may react to pain in three ways: trough vocalization, physiologically, and behaviorally [32, 33]. Thereby pain killers appear attractive in improving welfare for lame pigs. However, if analgesic treatment of lame piglets leads to an increased mobility during the acute lameness there might be a risk for overexertion of affected joints which in turn might induce long term negative side effects. Therefore it is important to note that no difference in weight gain between the two treated groups were recorded in this study. NSAID –treated piglets did not grew faster than non-NSAID-treated piglets, but neither did they grew slower which would have been expected if long term negative side effects would have been at hand. Use of NSAIDs in combination with antibiotics as treatment for lameness in piglets ought to therefore be considered for animal welfare issues, at least at severe cases of lameness.

In this study the clinical response of treatment penicillin was good, regardless of a similar treatment with NSAID or not. In both groups no piglet were given diagnose codes 0 or 1 (healthy or almost healthy) when initiating treatment, but 50 % of them were scored with 0 or 1 after 5 days of treatment. Yet it must be remembered that every 4^th^ piglet diagnosed with severe lameness (diagnose code 3) when treatment was initiated still had that clinical score day 5, and that piglets diagnosed with lameness had a reduced weight gain. Since an early initiated treatment is concluded to be essential for a good treatment prognosis [16, 34], persisting clinical score might mirror the time for initiation of treatment in relation to the true onset of infection. It should also be emphasized that reducing pain pharmacologically in lame piglets not can replace the management routines, floor quality and good care.

Although lameness most commonly was observed in back hoofs, followed by elbows, front hoofs and hocks, lameness was fairly evenly distributed between joints. This indicated a septicemial spread of the infections associated with lameness, as previously also indicated by the association to abrasions [26]. We have no explanation for the diverging distribution between the left (56.8 %) and the right (43.2 %) side of the pig, but similar observations have previously been reported from 264 preweaning piglets in England where 42 % had abrasions on the left limbs and 38 % on the right limbs [35].

The microbial cause of lameness in piglets may vary and treatment of lame pigs leads to a permanent use of antibiotics in piglet production, which in turn may lead to antimicrobial resistance. Therefore, a causative diagnose, including defining of minimum inhibitory concentration (MIC) values, ought to regularly be made from joints of lame piglets in pig herds. In this herd, bacterial cultivations revealed *Streptococcus dysgalactiae* subsp. *equisimilis* and *Staphylococcus hyicus* subsp. *hyicus* as the cause of infectious arthritis, which concur with several other reports [6, 8], that were sensitive to all antibiotics included in the antimicrobial panels used.

Prompt treatment with antibiotics of piglets that limp due to infection is required to achieve a positive treatment effect [1, 36] and in agreement with the results obtained in this study many studies recommend penicillin as first choice of antibiotics [34, 37, 38]. It could be argued that the statement on immediate treatment of lame piglets would increase use of antibiotics, but it should be remembered that no “just-to-be-sure strategic disease preventing antimicrobial treatment” whatsoever take place in any Swedish herd. This is a responsible way to ensure a low use of antimicrobials, based on the fact that healthy pigs do not need antibiotics.

## Conclusions

Piglets diagnosed with lameness had a reduced weight gain. Lameness was fairly evenly distributed between joints, which indicate a septicemic spread of the infections associated with lameness, as previously also indicated by the association to abrasions. The clinical response of penicillin was good, and it was neither improved nor reduced by a concurrent administration of NSAIDs. A significant finding of this study was that decreasing pain due to lameness not was negative in a long term perspective, *i.e.* reducing pain did not lead to overstrain of affected joints and no clinical signs of adverse effects were noted. Therefore the use of NSAIDs ought to be considered to improve the animal welfare, at least in severe cases.

Despite the generally good effect of penicillin, it was notable that around every 4^th^ piglet diagnosed with severe lameness still scored so 4 days after initiating treatment. Since inserting treatment during the early cause of joint diseases has been suggested to be important for a good prognosis this may have been dependant on the onset of treatment in relation to the true onset of infection.

## Methods

The study has been approved by the Ethical Committee for Animal Experiments, Uppsala, Sweden (reference number C 135/9). All lame piglets in the study would have been subjected to treatments with penicillin as a routine procedure regardless of the study, but none with NSAIDs.

### Animals and management routines

The study was carried out at research station at Funbo-Lövsta, Swedish University of Agricultural Sciences. The farrow-to-finish herd comprised 110 sows (mainly purebred Yorkshire) and had been established for 30 years. The herd was free from diseases according to the “A List” of the Office International des Epizooties, and also from Aujeszky’s disease, atrophic rhinitis, *Brachyspira* (*Serpuliana*) species, porcine epidemic diarrhoea, porcine reproductive and respiratory syndrome, *Salmonella* species and transmissible gastroenteritis.

Pregnant sows were housed in a deep-litter system in groups of about 16, but with individual feeding. Two weeks before farrowing, sows were transferred to a cleaned farrowing unit with 16 pens, each 8.4 m^2^ in area and bedded with straw. Each piglet was weighed and given an identity (tattoo) at the day of birth. Navels were disinfected, canine teeth were filed when judged necessary and, canvas was glued to carpal joints to prevent abrasions. The piglets were also weighed when they were 5 weeks (at weaning) and 9 weeks old.

All 6,787 piglets born alive during a period of two and a half years were included in the study. The male piglets were castrated at 2, 3 or 4 days of age, and at the same time all the piglets received an intramuscular injection of 200 mg iron as iron dextran (Pigeron; Leo Pharmaceutical). The piglets were given a second iron injection when they were 14 days old, and they were offered commercial creep feed without antibiotics from 3 weeks of age; it contained 15.5 % crude protein, 1.0 % lysine, and 12.2 MJ metabolisable energy (ME)/kg (Växfor; Lantmännen, Svalöv, Sweden). No routine strategical treatment with antimicrobials whatsoever were made in the herd. Only pigs diagnosed with a disease were medically treated.

### Lameness, treatment, necropsies, culture of bacteria, antimicrobial resistance

The herd veterinarian had made a written instruction to the staff. According to that instruction, lame piglets or piglets with visibly swollen joints were defined to suffer from lameness and should be parenterally treated with antimicrobials immediately. Benzyl penicillin (Penovet^®^ vet., Boehringer Ingelheim Vetmedica) was intramuscularly administered at a dose of 20 mg per kg bodyweight once a day for 5 days. Every second piglet was additionally injected with 3 mg ketoprofen (Romefen vet., Merial Norden) per kg bodyweight once a day for 3 days. Every medically treated piglet was colour-marked, and records of diseases and treatments were kept for each piglet. The staff was instructed to treat piglets affected by arthritis as early as possible to attain a fair treatment prognosis.

Three randomly selected lame piglets were culled instead of medically treated. At necropsy, samples for bacteriology were collected with sterile cotton swabs from up to 3 joints diagnosed with arthritis and from a normal joint from each pig. The samples were spread directly to blood agar (blood agar base No. 2; LabM, Salford, England + 5 % horse blood) and bromcresol purple-lactose agar (NVI art No.341200). The plates were incubated at 37 °C and read after 18 and 48 h. Isolates of staphylococci and streptococci were typed with methods used at the Bacteriological diagnostic laboratory at the National Veterinary Institute (NVI).

Isolates of staphylococci and streptococci were tested with respect to antimicrobial resistance towards penicillin, cephalothin, oxacillin +2 % NaCl, erythromycin, chloramphenicol, clindamycin, tetracycline, fusidic acid, gentamicin, kanamycin, ciprofloxacin, trimethoprim [VetMIC™ GP-mo-A (version 2), NVI].

### Clinical examinations and evaluation of therapeutic efficacy

The occurrence of lameness was registered from birth until the age of 5 weeks and the clinical efficacies of treatment were assessed daily. When diagnosed lame, pigs were given a clinical score based on lameness swollen joints and general health status; 0 = healthy; 1 = almost healthy; 2 = manifest lameness; 3 severe lameness.

The occurrence of lameness and affected joints (Elbow, Carpus, Hock, Metacarpal joint, Hoof) in one or more legs were registered from birth until the age of 5 weeks.

### Statistical analysis

Statistical analyses were performed using the Statistical Analysis Systems; SAS 9.2 (SAS, 2014). Data from the 6,787 liveborn piglets were included in the statistical analyses. Only the first incidence of arthritis in each piglet was taken into account and only complete recordings was included in the analyses. Differences in arthritis prevalence between sexes (male or female) and sow parities (1, 2, 3, 4, >4), as well as differences in clinical lameness status between treatments and change in clinical lameness status day to day was analysed in bivariate two-by-two chi square tests using PROC FREQ. This procedure enabled pairwise competitions of prevalences between all classes (e.g. between specific parities). Differences in weight between pigs treated for arthritis and unaffected pigs were analysed with MODEL 1 and 2 and between lame pigs given the two different treatments with MODEL 3 and 4 using PROC MIXED.

MODEL 1: Birth weight = Lameness (yes or no) + Sex (Male or Female) + Parity (1, 2, 3, 4 or >4) + Sow + Sow*Parity + e

MODEL 2: Weight at 5 weeks or Weight at 9 weeks = Lameness (yes or no) + Sex (Male or Female) + Parity (1, 2, 3, 4 or >4) + Sow + Sow*Parity + Birth weight + e

MODEL 3: Birth weight = Treatment (Penicillin + NSAID or Penicillin) + Sex (Male or Female) + Parity (1, 2, 3, 4 or >4) + Sow + Sow*Parity + e

MODEL 4: Weight at 5 weeks or Weight at 9 weeks = Treatment (Penicillin + NSAID or Penicillin) + Sex (Male or Female) + Parity (1, 2, 3, 4 or >4) + Sow + Sow*Parity + Birth weight + e

Where Lameness, Treatment, Sex and Parity were included as fixed effects, Sow and Sow*Parity were included as random effects and Birth weight was included as a continuous covariate.
